# Individual and community-level income and the risk of diabetes rehospitalization among women and men: a Canadian population-based cohort study

**DOI:** 10.1186/s12889-020-8159-1

**Published:** 2020-01-14

**Authors:** Neeru Gupta, Dan L. Crouse, Adele Balram

**Affiliations:** 1 Department of Sociology, University of New Brunswick, P.O. Box 4400, Fredericton, New Brunswick E3B 5A3 Canada; 2 New Brunswick Institute for Research, Data and Training (NB-IRDT), P.O. Box 4400, Fredericton, New Brunswick E3B 5A3 Canada

**Keywords:** Hospital admissions, Diabetes mellitus, Preventable hospitalization, Rehospitalization, Gender and ethnic differences, Low income

## Abstract

**Background:**

Marked disparities by socioeconomic status in the risk of potentially avoidable hospitalization for chronic illnesses have been observed in many contexts, including those with universal health coverage. Less well known is how gender mediates such differences. We conducted a population-based cohort study to describe associations between household and community-level income and rehospitalizations for types 1 and 2 diabetes mellitus among Canadian women and men.

**Methods:**

Our cohorts were drawn from respondents to the 2006 mandatory long-form census linked longitudinally to 3 years of nationally standardized hospital records. We included adults 30–69 years hospitalized with diabetes at least once during the study period. We used logistic regressions to estimate odds ratios for 12-month diabetes rehospitalization associated with indicators of household and community-level income, with separate models by gender, and controlling for a range of other sociodemographic characteristics. Since diabetes may not always be recognized as the main reason for hospitalization, we accounted for disease progression through consideration of admissions where diabetes was previously identified as a secondary diagnosis.

**Results:**

Among persons hospitalized at least once with diabetes (*n* = 41,290), 1.5% were readmitted within 12 months where the initial admission had diabetes as the primary diagnosis, and 1.8% were readmitted where the initial admission had diabetes as a secondary diagnosis. For men, being in the lowest household income quintile was associated with higher odds of rehospitalization in cases where the initial admission listed diabetes as either the primary diagnosis (OR = 2.21; 95% CI = 1.38–3.51) or a secondary diagnosis (OR = 1.51; 95% CI = 1.02–2.24). For women, we found no association with income and rehospitalization, but having less than university education was associated with higher odds of rehospitalization where diabetes was a secondary diagnosis of the initial admission (OR = 1.88; 95% CI = 1.21–2.92). We also found positive, but insignificant associations between community-level poverty and odds of rehospitalization.

**Conclusions:**

Universal health coverage remains insufficient to eliminate socioeconomic inequalities in preventable diabetes-related hospitalizations, as illustrated in this Canadian context. Decision-makers should tread cautiously with gender-blind poverty reduction actions aiming to enhance population health that may inadequately respond to the different needs of disadvantaged women and men with chronic illness.

## Background

Diabetes-related hospitalizations are often referred to as ambulatory care sensitive, that is, preventable and potentially avoidable through appropriate management in primary and community-based care [1–3]. Among patients with type 1 and type 2 diabetes, higher rates of hospitalization are often considered as a marker of poorer access to primary care and of inequity and inefficiency in the healthcare system in managing chronic conditions [2, 3]. In the United States and elsewhere, low income and insurance status have been linked to reduced access to preventive services for chronic conditions and, in turn, higher rates of hospitalization and rehospitalization [2, 4]. Even in countries of publicly funded universal health coverage, such as Canada, where essential medical and hospital services are provided based on need rather than on ability to pay, lower-income individuals have been found to be significantly more likely to experience a potentially avoidable hospitalization for diabetes and other chronic conditions compared to those in higher income groups [3, 5, 6]. Differences in the risk of preventable hospitalization for chronic disease by racial/ancestral group have been found in many contexts [7–9]. Women have also been found to experience greater increases in population-adjusted hospitalization rates for diabetes compared to men in the United States, possibly related to women catching up to their male counterparts in diabetes prevalence, leading to a shift in the hospital burden of diabetes [10].

Although policies and programs to strengthen timely access and use of ambulatory care should, in theory, prevent the onset of complications requiring acute inpatient care and thereby reduce overall healthcare costs, the evidence on which levers actually work to promote this agenda at the individual and population levels remains inconclusive.

Identified strategies to reduce the number of potentially avoidable hospitalizations for chronic illness are numerous, and may include patient, provider, medical, social, informational, and system-related characteristics [11–13]. Management continuity is often stressed as crucial to reducing the risk of hospitalization or rehospitalization, but there is little evidence-informed consensus on the definition of continuity of care, most effective and efficient approach, or most appropriate related outcomes to measure [11]. This information gap is attributable in part to a paucity of studies that have been able to consider a wide range of potential confounders, including patients’ general health status [1]. Relatively few studies provide policy-actionable evidence on the medical and non-medical reasons as to why certain population groups may be more vulnerable to experience avoidable hospitalization, regardless of universal health care, and how gender might play a substantial role.

The purpose of this study is to contribute to the evidence base on income inequalities and other social determinants of potentially avoidable rehospitalization for diabetes to inform primary care and public health strategies for prevention. We focus on the risk of 12-month diabetes rehospitalization; this measure lends to practical approaches to incorporating health status given the most commonly available data in many population health and health services research settings, is a better metric to identify achievable targets, is less sensitive to shorter term hospital-level quality of care concerns, is more likely to capture readmissions due to disease progression, and is more reflective of care continuity regardless of whether the patient sees the same or a different provider [11, 14], thereby presenting it as the low-hanging fruit for diabetes management assessment.

Specifically, we analyse the 2006 Canadian Census-Discharge Abstract Database (DAD) linked data, a unique large-scale resource (4.65 million long-form census respondents and 3 million inpatient records annually) to support investigations of hospital use by sub-groups and socioeconomic status. We use a prospective cohort approach to provide the first comprehensive national assessment of gendered differences in diabetes rehospitalizations associated with indicators of both household- and community-level income. We hypothesize that lower household and community-level incomes are independently associated with increased risk of 12-month diabetes rehospitalization among Canadian adults. We further hypothesize that these differentials are mediated by gender, lending actionable insights for targeting health and social investments to lower the number of hospital readmissions through enhanced equity.

## Methods

Drawing on linked population-based and administrative health data sets, our study conforms to the STROBE (STrengthening the Reporting of OBservational studies in Epidemiology) protocol [15].

### Study setting

Across Canada, an estimated 7.7% of the population aged 1 year and older are living with type 1 or type 2 diabetes [16]. Prevalence is higher among males (8.3%) than females (7.2%), and increases with age. The age-standardized prevalence of diabetes rose at an average annual rate of 4.2% between 2000 and 2011, a trend attributable in part to rising rates of obesity and overweight [16]. While the *Canada Health Act* ensures that essential hospital and physician services are covered for all residents through public revenues, gaps remain in the equitable access to certain services [17]. For one, it is has been estimated that persons with diabetes in higher household income groups were more likely to receive the recommended care to prevent complications (such as an HbA1c test, a urine test for protein, a dilated eye exam, and a foot exam) compared to those with the lowest household income, after adjusting for age [18]. A recent national study on hospitalized diabetes underscored the importance of using a sex-based analysis: while more males (58.7%) were counted among those hospitalized for diabetes as the most responsible diagnosis, differing patterns were seen among females for selected comorbidities [19]. Combining data from multiple sources is required is better understand the subtleties of risk and protective factors for potentially avoidable diabetes-related hospitalizations.

### Data sources

We used data collected in the 2006 mandatory long-form census [20] linked to nationally standardized data on inpatient care for the fiscal years 2006/07 to 2008/09 obtained from the hospital Discharge Abstract Database [21]. The 2006 long-form census questionnaire gathered data from one in five Canadian households representing 95 to 97% of the population across provinces (accounting for population net undercoverage). Information was collected for all household members (4.65 million individuals) on a range of topics such as income, education, ethnicity, and household living arrangements. The DAD collates basic demographic, administrative, and clinical data for approximately 3 million hospital stays each year, covering virtually all acute-care and some psychiatric, chronic, rehabilitation, and day-surgery discharges in nine of the ten provinces (except Quebec). As detailed elsewhere [22], hierarchical deterministic exact matching was employed to link census with hospital data, based on linkage keys derived from three variables common to both data sources: sex, date of birth, and postal code. Since each DAD record corresponds with one hospitalization, individuals with multiple hospitalizations will have multiple records.

### Cohort identification

Our cohort comprised women and men ages 30–69 years at the time of the census who were hospitalized at least once during the period of observation with a recorded diagnosis of type 1 or type 2 diabetes mellitus, as classified in the administrative data by the Canadian adaptation of the *International Statistical Classification of Diseases and Related Health Problems, 10th Revision* (ICD-10), codes E10.x-E14.x [23]. Validity of diabetes diagnoses in the DAD has been assessed to be high (sensitivity > 81%, specificity > 93%, and positive predictive value > 81%) [24]. Excluded from the cohort analysis were patients without any diagnosis of diabetes or who died during their first hospital stay with identified diabetes, because these subjects would not have had an opportunity to have a diabetes readmission.

### Outcome measures

We searched the linked hospital records prospectively over the three-year period to capture characteristics of each individual’s first hospitalization with identified diabetes, referred to as the index admission. Since diabetes may not always be recognized as the main reason for hospitalization, we distinguished two exposures: where the index admission had a primary diagnosis of diabetes, that is, with the condition being considered most responsible for the patient’s hospital stay (i.e., listed first of the potential 25 diagnostic codes in the record); and where the index admission had a secondary diagnosis of diabetes, that is, for which the patient may or may not have received treatment, but which was assigned a diagnostic code (i.e., diagnostic fields 2 through 25). We then defined two outcomes: readmission to any hospital at least once within 12 months with diabetes as primary diagnosis by whether the index admission had diabetes as (i) the primary diagnosis or (ii) a secondary diagnosis.

### Key covariates

Our main predictors of interest were individual- and community-level income adequacy, as measured in the census. Low income status (or poverty status) is among the most frequently examined indicators of socioeconomic status, and is associated with multiple risk factors for diabetes and its complications, but few studies have examined the relation of both individual and community poverty measures with chronic disease outcomes [25]. Individual income levels were assessed in this study from the distribution by quintiles of the sum of after-tax incomes received in the household during the preceding year from all sources, adjusted for household size. This approach was considered an appropriate measure of funds available, since income is often pooled within a household [26]. We further studied the effects of community-level income. We dichotomized community income status, defining low-income communities as those in which 30% or more of residents fell below an established low-income measure [27, 28]. Here we defined community as census subdivisions, which are roughly equivalent in size to a municipality or rural community.

### Statistical analysis

We used multiple logistic regression to estimate odds ratios (ORs) and corresponding 95% confidence intervals (CIs) associated with 12-month diabetes rehospitalization by index admission. In addition to considering the influences of household and community income levels, we controlled for a number of sociodemographic characteristics widely related in the literature with differential diabetes-related health and healthcare outcomes: sex, age (in single years among individuals aged 30–69), marital status, Aboriginal identity, immigrant status, and educational attainment [3, 6, 7, 29]. Although there was information specific to each hospitalization over the three-year period, the individual and contextual characteristics were captured only at baseline (from the 2006 census). To see if there was a gender difference in the socioeconomic correlates of rehospitalization risks, we ran models for men and women separately. The models were further stratified by province of residence to account for interjurisdictional health policy and planning contextual specificities (grouping the two smallest neighbouring provinces to ensure minimum sample sizes of rehospitalizations). Analyses were conducted using the SAS 9.4 statistical software package. Population counts were rounded to a base of five, and adjusted to reinforce the confidential nature of the data using control algorithms specified by the national statistical agency. All estimates were calibrated using person-level census weights to ensure population representation [20].

## Results

Following the linkage of the census and hospital administrative data sets, our cohort included 41,290 adults (females: 18,330; males: 22,960) ages 30–69 years hospitalized with diagnosed diabetes at least once over the study period. Males were over-represented among those ever hospitalized with diabetes, a pattern generally reflecting established epidemiological patterns especially in populations with higher diabetes incidence [30]. Within the study cohort, 1.5% (610 patients) were readmitted within 12 months for diabetes complications where the index hospitalization had diabetes as primary diagnosis, and 1.8% (750 patients) were readmitted where the index hospitalization had diabetes as secondary diagnosis. Among the latter, nearly one in five (18%) had initially been hospitalized for a chronic ambulatory care sensitive condition that was concordant with diabetes care, such as hypertension or ischemic heart disease (not shown).

Table 1 presents a descriptive analysis by characteristics of the individuals rehospitalized for diabetes. Where the index hospitalization had diabetes as primary diagnosis, rehospitalizations were less common among women than men (1.2% versus 1.7%) and among older adults ages 50–69 years compared to those 30–39 years (~ 1.1% versus 2.9%). Conversely, where the index hospitalization had diabetes as a secondary diagnosis, rehospitalizations were somewhat less common among men and among those ages 40–69. Regardless of the most responsible diagnosis for the index hospitalization, as expected, those of higher household income, who were currently married or in union, had a university degree, were foreign-born, of non-Aboriginal identity, or were living in communities of higher income levels experienced lower rates of 12-month diabetes rehospitalization than the general population-based cohort.

**Table 1:** Percent who experienced a 12-month diabetes rehospitalization among the population 30–69 years ever hospitalized with diabetes, by diagnostic information for the index admission, according to sociodemographic characteristics, Canada, 2006–2008

Characteristic	Index hospitalization with a primary diagnosis of diabetes	Index hospitalization with a secondary diagnosis of diabetes
Sex		
Female	1.2%	1.9%
Male	1.7%	1.8%
Age group		
30–39 years	2.9%	2.3%
40–49 years	2.4%	1.7%
50–59 years	1.2%	1.9%
60–69 years	1.1%	1.7%
Household income quintile		
Lowest	1.9%	2.2%
Lower-middle	1.4%	1.9%
Middle	1.3%	1.5%
Middle-upper	1.1%	1.6%
Highest	1.0%	1.2%
Marital status		
Currently married or in union	1.2%	1.6%
Formerly married or in union	1.5%	2.2%
Never married or in union	2.6%	2.1%
Educational attainment		
Less than university diploma	1.6%	1.9%
University diploma or degree	1.0%	1.2%
Immigrant status		
Native-born	1.7%	2.0%
Immigrant	0.8%	1.2%
Aboriginal identity		
Non-Aboriginal identity	1.2%	1.5%
Aboriginal identity	2.7%	3.3%
Living in a low-income community		
Yes	2.6%	3.2%
No	1.3%	1.6%
Total	1.5%	1.8%

Note: Data are based on a cohort of 41,290 patients hospitalized at least once with diagnosed diabetes (type 1 or type 2) over the three-year study period. Characteristics are those at the time of the census. Residents of Quebec and the territories are excluded

Source: 2006 Census linked to 2006/07–2008/09 Discharge Abstract Database

Results from the multiple regression models show that, among adult women, household income was not significantly associated with the risk of rehospitalization, after controlling for other sociodemographic characteristics, and regardless of the most responsible diagnosis for the index hospitalization (Table 2). Increasing years of age was among the factors associated with significantly lower odds of rehospitalization where diabetes was the primary diagnosis of the index hospitalization (OR = 0.97; 95% CI = 0.95–0.98) (Table 2, Model 1). Women of Aboriginal identity had significantly greater odds of rehospitalization compared to non-Aboriginal women (OR = 1.55; 95% CI = 1.12–2.15), all else being equal. Where diabetes was a secondary diagnosis of the index hospitalization, the risk of rehospitalization was significantly greater among those with less than university educational attainment (OR = 1.88; 95% CI = 1.12–2.92) and among those of Aboriginal identity (OR = 1.94; 95% CI = 1.49–2.52) (Table 2, Model 3).

**Table 2:** Adjusted odds-ratios (and 95% confidence intervals) from the multiple logistic regression models for the risk of 12-month diabetes rehospitalization among the female population 30–69 years, by diagnostic information for the index admission

	Index hospitalization with a primary diagnosis of diabetes		Index hospitalization with a secondary diagnosis of diabetes	
	Model 1 (OR and 95% CI)	Model 2 (OR and 95% CI)	Model 3 (OR and 95% CI)	Model 4 (OR and 95% CI)
Age (years)	0.97* (0.95–0.98)	0.97* (0.95–0.98)	1.01 (1.00–1.02)	1.01 (1.00–1.02)
Household income quintile				
Lowest	0.95 (0.57–1.57)	0.95 (0.57–1.57)	1.12 (0.69–1.81)	1.10 (0.68–1.79)
Lower-middle	0.81 (0.48–1.37)	0.81 (0.48–1.37)	1.22 (0.75–1.98)	1.21 (0.74–1.97)
Middle	0.70 (0.39–1.23)	0.70 (0.39–1.23)	0.89 (0.52–1.52)	0.89 (0.52–1.51)
Middle-upper	0.56 (0.29–1.06)	0.56 (0.29–1.06)	1.13 (0.66–1.96)	1.13 (0.66–1.96)
Highest (ref)	1.00	1.00	1.00	1.00
Marital status				
Currently married or in union (ref)	1.00	1.00	1.00	1.00
Formerly married or in union	1.24 (0.90–1.71)	1.24 (0.90–1.71)	1.17 (0.91–1.50)	1.18 (0.91–1.51)
Never married or in union	0.99 (0.67–1.46)	0.99 (0.67–1.46)	1.08 (0.77–1.51)	1.08 (0.77–1.51)
Educational attainment				
Less than university diploma	1.12 (0.72–1.73)	1.12 (0.72–1.73)	1.88* (1.21–2.92)	1.88* (1.21–2.92)
University diploma or degree (ref)	1.00	1.00	1.00	1.00
Immigrant status				
Native-born (ref)	1.00	1.00	1.00	1.00
Immigrant	0.87 (0.58–1.31)	0.87 (0.58–1.31)	0.88 (0.63–1.23)	0.88 (0.63–1.23)
Aboriginal identity				
Non-Aboriginal identity (ref)	1.00	1.00	1.00	1.00
Aboriginal identity	1.55* (1.12–2.15)	1.51 (0.99–2.31)	1.94* (1.49–2.52)	1.79* (1.27–2.52)
Living in a low-income community				
Yes	–	1.04 (0.67–1.62)	–	1.14 (0.80–1.62)
No (ref)	–	1.00	–	1.00

Note: * = *p* < 0.05; ref. = reference category. Data are based on 18,330 female patients hospitalized at least once with diagnosed diabetes (type 1 or 2) over the study period. Models 1 and 3: odds-ratios adjusted for household income quintile and other individual-level sociodemographic characteristics; Models 2 and 4 additionally adjusted for patients’ community-level income. All models stratified by province of residence

Where diabetes was the primary diagnosis of the index hospitalization, men in the lowest income group had more than twice the odds of rehospitalization compared to those in the highest income quintile (OR = 2.21; 95% CI = 1.38–3.51), and nearly twice the odds in the lower-middle group (OR = 1.92; 95% CI = 1.20–3.09) (Table 3, Model 1). Significant protective factors for the risk of rehospitalization included increasing years of age (OR = 0.97; 95% CI = 0.96–0.98) and being foreign-born (OR = 0.55; 95% CI = 0.40–0.77). Men who had never been married were at greater risk (OR = 1.56; 95% CI = 1.20–2.03) as were men of Aboriginal identity (OR = 1.46; 95% CI = 1.13–1.88). Where diabetes was a secondary diagnosis of the index hospitalization, the risk of rehospitalization was significantly higher among those in the lowest household income quintile compared to the highest (OR = 1.51; 95% CI = 1.02–2.24), those who were widowed or divorced compared to those currently married (OR = 1.31; 95% CI = 1.02–1.67), and those of Aboriginal identity (OR = 1.81; 95% CI = 1.40–2.33) (Table 3, Model 3).

**Table 3:** Adjusted odds-ratios (and 95% confidence intervals) from the multiple logistic regression models for the risk of 12-month diabetes rehospitalization among the male population 30–69 years, by diagnostic information for the index admission

	Index hospitalization with a primary diagnosis of diabetes		Index hospitalization with a secondary diagnosis of diabetes	
	Model 1 (OR and 95% CI)	Model 2 (OR and 95% CI)	Model 3 (OR and 95% CI)	Model 4 (OR and 95% CI)
Age (years)	0.97* (0.96–0.98)	0.97* (0.96–0.98)	0.99* (0.98–1.00)	0.99* (0.98–1.00)
Household income quintile				
Lowest	2.21* (1.38–3.51)	2.19* (1.37–3.49)	1.51* (1.02–2.24)	1.48 (1.00–2.20)
Lower-middle	1.92* (1.20–3.09)	1.91* (1.19–3.08)	1.39 (0.93–2.08)	1.38 (0.93–2.06)
Middle	1.72* (1.05–2.81)	1.72* (1.05–2.80)	1.23 (0.81–1.86)	1.22 (0.80–1.86)
Middle-upper	1.64 (0.98–2.73)	1.64 (0.98–2.72)	1.31 (0.85–2.02)	1.31 (0.85–2.01)
Highest (ref)	1.00	1.00	1.00	1.00
Marital status				
Currently married or in union (ref)	1.00	1.00	1.00	1.00
Formerly married or in union	1.11 (0.84–1.46)	1.11 (0.84–1.47)	1.31* (1.02–1.67)	1.31* (1.03–1.68)
Never married or in union	1.56* (1.20–2.03)	1.56* (1.20–2.03)	0.90 (0.67–1.21)	0.90 (0.67–1.22)
Educational attainment				
Less than university diploma	1.34 (0.93–1.94)	1.34 (0.93–1.94)	1.20 (0.87–1.67)	1.20 (0.87–1.67)
University diploma or degree (ref)	1.00	1.00	1.00	1.00
Immigrant status				
Native-born (ref)	1.00	1.00	1.00	1.00
Immigrant	0.55* (0.40–0.77)	0.55* (0.40–0.78)	0.72* (0.54–0.96)	0.72* (0.54–0.96)
Aboriginal identity				
Non-Aboriginal identity (ref)	1.00	1.00	1.00	1.00
Aboriginal identity	1.46* (1.13–1.88)	1.39* (0.99–1.95)	1.81* (1.40–2.33)	1.61* (1.15–2.25)
Living in a low-income community				
Yes	–	1.08 (0.76–1.54)	–	1.21 (0.85–1.71)
No (ref)	–	1.00	–	1.00

Note: * = *p* < 0.05; ref. = reference category. Data are based on 22,960 male patients hospitalized at least once with diagnosed diabetes (type 1 or 2) over the study period. Models 1 and 3: odds-ratios adjusted for household income quintile and other individual-level sociodemographic characteristics; Models 2 and 4 additionally adjusted for patients’ community-level income. All models stratified by province of residence

Further adjusting for community deprivation, in all models for women and men (Models 2 and 4), there was suggestion of increased odds of rehospitalization associated with community-level poverty, but none of these results were statistically significant (e.g., OR = 1.21 [0.85–1.71] for men where diabetes was a secondary diagnosis of the index hospitalization, as seen in Table 3, Model 4).

## Discussion

Research suggests that socioeconomic status is associated with a myriad of long- and short-term complications of type 1 and type 2 diabetes in adults, including poorer glycemic control and greater morbidity and mortality, and this regardless of the health system financing arrangements [31, 32]. Our population-based cohort analysis using linked census and administrative data in the Canadian context of universal health coverage revealed that, among adult men, being in the lowest household income quintile was significantly associated with higher risk of 12-month rehospitalization, in cases where the index hospitalization listed diabetes either as the primary diagnosis (OR = 2.21; 95% CI = 1.38–3.51) or as a secondary diagnosis (OR = 1.51; 95% CI = 1.02–2.24), a pattern that generally held when further controlling for community poverty. Among adult women, the measure of socioeconomic status that was found to exercise an independent effect was below university educational attainment, associated with significantly higher risk of rehospitalization where diabetes was a secondary diagnosis of the index hospitalization (OR = 1.88; 95% CI = 1.21–2.92), results that held when controlling for community poverty.

The results of this study raise caution that “gender-blind” poverty reduction strategies aiming to enhance population health may have intended consequences on gendered health outcomes. It is possible, for example, that public investments in family income supplementation may yield better results (i.e., fewer hospital readmissions) among adult males, but may inadvertently contribute to gender gaps by inadequately responding to the needs of disadvantaged women with multiple morbidities. Differential impacts of income by gender were also previously found in a study of the risk of diabetes-related amputation in a Canadian province, with low income found to be a less heightened risk factor among women than men despite similar use of primary care services [33]. On the other hand, we found little relation of community-level poverty with the risk of 12-month rehospitalization. This suggests that interventions to mitigate socioeconomic disparities in severe complications of diabetes may be more effectively approached at the individual level, a finding consistent with the conclusions of a study on the influences of individual and community poverty on risk of end stage renal disease among adults in the United States [25]. Small numbers of rehospitalization events may also have limited the statistical power of our models to tease significant associations.

Although population aging is often associated with concerns for sustainability of the healthcare system in managing chronic diseases, our results showed that the risk of rehospitalization decreases with age where diabetes was the primary diagnosis of the initial hospitalization, and this for both females (OR = 0.97; 95%CI = 0.95–0.98) and males (OR = 0.97; 95%CI = 0.96–0.98). The significance of the relationship did not hold when diabetes was a secondary diagnosis of the index hospitalization. This may reflect a differential risk among adult patients with type 1 diabetes versus those with type 2 diabetes, the latter being more likely to present comorbid conditions but for whom diabetes was not necessarily identified as the cause of hospitalization. Limitations of the ICD-10 coding standard to consistently distinguish type 1 from type 2 diabetes using administrative databases hindered our ability to investigate this dynamic further. Associations between other sociodemographic indicators and risk of diabetes rehospitalization generally followed the expected patterns. The protective nature of being an immigrant in Canada was seen, widely attributed to the “healthy immigrant effect” stemming from systematic medical screenings and positive self-selection [29]. Our results were also consistent with previous investigations suggesting Aboriginal identity is associated with increased risk of hospitalization, but which relied on proportional area-based measures of Aboriginal populations given the lack of consistent classification of Aboriginal identity in Canadian administrative health data sets [7].

The linking of population survey data with administrative health records has greatly opened opportunities for enhanced research on the social determinants of health and diabetes compared to previous analyses using administrative data alone [34, 35]. However, a certain underestimate of the number of diabetes rehospitalizations captured in this study is likely. Patients may have experienced multiple hospitalizations over the 3 years of observation. We chose to measure the outcome dichotomously, thereby excluding repeat diabetes-related admissions during this period. In addition, 12-month readmissions would have been right-censored in the last of the 3 years of data. The analysis was limited to patients who survived their first hospital episode, since otherwise they would not have run the risk of readmission, but this excludes hospital mortality as a potential measure of quality of care. Because the hospital data excluded records for Quebec, not only are we missing hospitalizations for residents of this province, but also an unknown number of readmissions among residents in border communities in its neighbouring provinces (that is, New Brunswick, Newfoundland and Labrador, and Ontario). In the Canadian context, the federal act governing universal health care ensures portability of provincial health insurance anywhere in the country.

Despite the limitations noted above, this study represents, to the best of our knowledge, the first Canadian population-wide gender-based cohort analysis of individual- and community-level income and other sociodemographic factors associated with diabetes-related rehospitalizations. We were able to tap into the rich information available uniquely through the population census linked to cause-specific data on hospital stays. While our data source lacked information on diabetes diagnoses in primary care and on behavioural risk factors amenable to public health intervention, we accounted for disease progression through examination of the main reason for each index hospitalization, a unique approach to better understand the factors influencing continuity of care where diabetes was a primary versus secondary reason for the initial admission.

## Conclusions

Results from our population-based cohort analysis on the drivers of diabetes rehospitalizations among Canadians aged 30–69 emphasized divergences by indicators of socioeconomic status, and substantiated that universal health coverage remains insufficient to eliminate inequalities in adverse diabetes-related health outcomes. The results further amplified the need to consider factors beyond individual income adequacy to distinguish gender differences in terms of which measures of socioeconomic status are most amenable to policy actions to reduce potentially avoidable diabetes hospitalizations. Our findings reinforce the urgent need for better integration between levels of care for adult patients with diabetes to minimize hospital admissions, focusing on the persistent differential social risk and protective factors among women and men.

## Data Availability

The data that support the findings of this study are available through Statistics Canada’s Research Data Centres but restrictions apply to the availability of these confidential data, which were used with permission for the current study, and so are not publicly available.

## Abbreviations

CI: Confidence interval
DAD: Discharge Abstract Database
ICD-10: International Classification of Diseases, 10th Revision
OR: Odds ratio
